# Diagnostic properties of differing BP thresholds for adverse pregnancy outcomes in standard-risk nulliparous women: A secondary analysis of SCOPE cohort data

**DOI:** 10.1371/journal.pmed.1004471

**Published:** 2025-01-22

**Authors:** Laura Slade, Maya Blackman, Hiten D. Mistry, Jeffrey N. Bone, Milly Wilson, Nuhaat Syeda, Lucilla Poston, Peter von Dadelszen, Laura A. Magee, SCOPE Consortium

**Affiliations:** 1 Robinson Research Institute, The University of Adelaide, Adelaide, Australia; 2 Department of Obstetrics and Gynaecology, Women’s and Children’s Hospital, Adelaide, Australia; 3 Department of Women and Children’s Health, School of Life Course and Population Sciences, Faculty of Life Sciences and Medicine, King’s College London, London, United Kingdom; 4 Department of Obstetrics and Gynaecology, University of British Columbia, Vancouver, Canada; 5 British Columbia Children’s Hospital Research Institute, University of British Columbia, Vancouver, Canada; Makerere University College of Health Sciences, UGANDA

## Abstract

**Background:**

In 2017, the American College of Cardiology and American Heart Association (ACC/AHA) lowered blood pressure (BP) thresholds to define hypertension in adults outside pregnancy. If used in pregnancy, these lower thresholds may identify women at increased risk of adverse outcomes, which would be particularly useful to risk-stratify nulliparous women. In this secondary analysis of the SCOPE cohort, we asked whether, among standard-risk nulliparous women, the ACC/AHA BP categories could identify women at increased risk for adverse outcomes.

**Methods and findings:**

Included were pregnancies in the international SCOPE cohort with birth at ≥20 weeks’ gestation, 2004 to 2008. Women were mostly of white ethnicity, in their 20s, and of normal-to-overweight body mass index (BMI). Excluded were pregnancies ending in fetal loss at <20 weeks’ gestation, and those terminated at any point in pregnancy. Women were categorized by highest BP during pregnancy, using ACC/AHA criteria: normal (BP <120/80 mmHg), “Elevated BP” (BP 120 to 129 mmHg/<80 mmHg), “Stage-1 hypertension” (systolic BP [sBP] 130 to 139 mmHg or diastolic BP [dBP] 80 to 89 mmHg), and “Stage-2 hypertension” that was non-severe (sBP 140 to 159 mmHg or dBP 90 to 109 mmHg) or severe (sBP ≥160 mmHg or dBP ≥110 mmHg). Primary outcomes were preterm birth (PTB), low birthweight, postpartum hemorrhage, and neonatal care admission. Adjusted relative risks (aRRs) and diagnostic test properties were calculated for each outcome, according to: each BP category (versus “normal”), and using the lower limit of each BP category as a cut-off. RRs were adjusted for maternal age, BMI, smoking, ethnicity, and alcohol use. Of 5,628 women in SCOPE, 5,597 were included in this analysis. When compared with normotension, severe “Stage 2 hypertension” was associated with PTB (24.0% versus 5.3%; aRR 4.88, 95% confidence interval, CI [3.46 to 6.88]), birthweight <10th centile (24.4% versus 8.8%; aRR 2.70 [2.00 to 3.65]), and neonatal unit admission (32.9% versus 8.9%; aRR 3.40 [2.59 to 4.46]). When compared with normotension, non-severe “Stage 2 hypertension” was associated with birthweight <10th centile (16.1% versus 8.8%; aRR 1.82 [1.45 to 2.29]) and neonatal unit admission (15.4% versus 8.9%; aRR 1.65 [1.31 to 2.07]), but no association with adverse outcomes was seen with BP categories below “Stage 2 hypertension.” When each BP category was assessed as a threshold for diagnosis of abnormal BP (compared with BP values below), only severe “Stage 2 hypertension” had a useful (good) likelihood ratio (LR) of 5.09 (95% CI [3.84 to 6.75]) for PTB. No BP threshold could rule-out adverse outcomes (i.e., had a negative LR <0.2). Limitations of our analysis include lack of ethnic diversity and use of values from clinical notes for BP within 2 weeks before birth. This study was limited by: its retrospective nature, not all women having BP recorded at all visits, and the lack of detail about some outcomes.

**Conclusions:**

In this study, we observed that 2017 ACC/AHA BP categories demonstrated a similar pattern of association and diagnostic test properties in nulliparous women, as seen in the general obstetric population. BP thresholds below the currently used “Stage 2 hypertension” were not associated with PTB, low birthweight, postpartum hemorrhage, or neonatal unit admission. This study does not support implementation of lower BP values as abnormal in nulliparous pregnant women.

## Abstract 2

Laura Slade, Maya Blackman and colleagues assess the associations between differing blood pressure thresholds and adverse pregnancy outcomes in a cohort of standard-risk nulliparous women.

## Author summary

**Why was this study done?:**

- In 2017, the American College of Cardiology (ACC) and American Heart Association (AHA) revised what they considered to be normal blood pressure (BP) outside pregnancy.
- No pregnancy guidelines have revised their definition of hypertension, which is a systolic blood pressure ≥140 mmHg or a diastolic blood pressure ≥90 mmHg, which ACC/AHA criteria outside pregnancy classify as “Stage 2 hypertension.”
- We performed a secondary analysis of the SCOPE pregnancy cohort, asking whether among standard-risk nulliparous women, the ACC/AHA BP categories before “Stage 2 hypertension” could identify women at risk for adverse pregnancy outcomes.

**What did the researchers do and find?:**

- For the available outcomes of preterm birth (PTB), birthweight <10th percentile, postpartum hemorrhage, and neonatal unit admission, only “Stage 2 hypertension” was associated with an increased risk of adverse outcomes.
- The association with the adverse outcomes evaluated was particularly true for severe “Stage 2 hypertension,” defined as a systolic blood pressure ≥160 mmHg or a diastolic blood pressure ≥110 mmHg.

**What do these findings mean?:**

- The findings of this study do not support implementation of lower BP values as abnormal in nulliparous pregnant women.
- Our findings are limited by a lack of ethnic diversity in our study population and use of clinical BP measurements for values within 2 weeks before birth.

## Introduction

International consensus defines hypertension in non-pregnant persons as a systolic blood pressure (sBP) ≥140 mmHg or a diastolic blood pressure (dBP) ≥90 mmHg [1,2]. When defined in this way, hypertension in pregnancy identifies women and babies at increased risk of adverse maternal, fetal, and neonatal outcomes [3]. These risks are widely recognized, whether BP is elevated before pregnancy or 20 weeks’ gestation (i.e., chronic hypertension) or at ≥20 weeks (i.e., gestational hypertension) [4,5]. This makes classification of hypertension in pregnancy and the management of abnormal BP crucial to minimize adverse outcomes.

In 2017, the American College of Cardiology/American Heart Association (ACC/AHA) altered their definitions of abnormal BP outside of pregnancy, given the continuous relationship between elevated BP and cardiovascular disease, the prevalence of which continues to rise [6,7]. Hypertension was reclassified by the ACC/AHA as “Stage 1 hypertension” (BP 130 to 139/80 to 89 mmHg) and “Stage 2 hypertension” (BP ≥140/90 mmHg), with “Elevated” BP (sBP 120 to 129 mmHg and dBP <80 mmHg) as an additional category before BP was considered to be “Normal” (<120/80 mmHg) [8].

A continuous relationship between BP levels in pregnancy and adverse outcomes has been demonstrated [9–11]; however, guidelines require a cutoff for risk delineation and appropriate resource utilization. Publications examining the relationship between the 2017 ACC/AHA BP cutoffs and adverse pregnancy outcomes have usually focused on BP measurements taken at <20 weeks’ gestation, as predictors of preeclampsia or other adverse outcomes [12–16], rather than the diagnostic test properties of the different cutoff levels. Few publications have focused specifically on nulliparous women [17,18], who are at higher risk of developing a hypertensive disorder of pregnancy (HDP), especially compared with multiparous women with a prior normotensive pregnancy.

While there have been calls to adopt the 2017 ACC/AHA BP categories in pregnancy [19], these have not yet been implemented in the American College of Obstetricians and Gynecologists (ACOG) guidelines or internationally [1]. In a cohort of standard-risk nulliparous women, we examined the potential benefits of using the 2017 ACC/AHA BP categories to identify women and babies at increased risk of preterm birth (PTB), small-for-gestational age (SGA) birthweight, postpartum hemorrhage (PPH), and neonatal intensive care unit (NICU) admission—outcomes for which hypertension is a known risk factor.

## Methods

### The SCOPE study

SCOPE (Screening for Pregnancy Endpoints) was an international, multicenter, prospective cohort study of standard-risk, nulliparous women with singleton pregnancies. The study aim was to develop screening tests to predict common pregnancy complications. The study was approved by the relevant local ethics committees (New Zealand AKX/02/00/364, Australia REC 1712/5/2008, London, Leeds and Manchester 06/MRE01/98 and Cork ECM5 (10) 05/02/08). The methods have been published previously [20].

In brief, from November 2004 to September 2008, potentially eligible women were approached to participate when they attended routine antenatal care at the 6 study sites: Auckland, New Zealand; Adelaide, Australia; London, Manchester and Leeds, UK; and Cork, Ireland. The inclusion criteria were: nulliparous women with a singleton pregnancy at 14^+0^ to 16^+6^ weeks’ gestation; these included women with a history of chronic hypertension that was not requiring antihypertensive therapy. Excluded were women: considered to be at high-risk for developing preeclampsia, PTB, or an SGA infant (including those with a history of chronic hypertension treated pre-pregnancy, women with preexisting diabetes or renal disease, systemic lupus erythematosus or anti-phospholipid syndrome); women with a major fetal anomaly; with BP ≥160/100 mmHg at booking for antenatal care; or who were taking low dose aspirin, calcium, fish oil, or heparin.

Women gave written, informed consent. The protocol involved 2 antenatal study visits at 14 to 16 and 19 to 21 weeks’ gestation and another visit within 48 h after birth. At each visit, BP was recorded by midwives, using devices validated for BP in pregnancy. BP was taken twice, with a mercury or aneroid Sphygmomanometer and then a MICROLIFE BP 3AC1-2 monitor at each study visit, and the second measurement from each recorded. Clinical data collected from antenatal clinic notes at each visit included demographic information, medical history, previous obstetric history, and family history of obstetric and medical disorders. Clinic notes for the mother and baby were reviewed for BP values recorded in the last 2 weeks of pregnancy prior to the onset of labor, as well as maternal and perinatal outcomes. Outcomes were recorded based on chart reviews and diagnostic codes, depending on what was available at the centers participating in this trial. These outcomes included gestational hypertension (i.e., systolic BP ≥140 mmHg or diastolic BP ≥90 mmHg in the second half of pregnancy), with or without preeclampsia, or preeclampsia specifically (defined as gestational hypertension plus proteinuria, including preeclampsia superimposed on chronic hypertension).

### BP and adverse pregnancy outcome

In this secondary analysis of SCOPE data, we examined the relationship between peak antenatal BP and adverse pregnancy outcomes associated with hypertensive pregnancy. We included pregnancies with BP values from at least 1 antenatal study visit, and data for at least one of our maternal or fetal/newborn outcomes. The project was approved by the SCOPE Research Committee, as authorized by the study’s original ethical approvals [21].

The highest antenatal BP was determined from values at the 3 time points available (i.e., 14 to 16 weeks, 19 to 21 weeks, and during the 2 weeks before birth), as part of the original SCOPE data collection. BP was classified according to the 2017 ACC/AHA guidelines, as follows: “Normal” BP (BP <120/<80 mmHg), “Elevated BP” (sBP 120 to 129 mmHg and dBP <80 mmHg), “Stage 1 hypertension” (sBP 130 to 139 mmHg or dBP 80 to 89 mmHg), and “Stage 2 hypertension” that was non-severe (sBP 140 to 159 mmHg or dBP 90 to 109 mmHg) or severe (sBP ≥160 mmHg or dBP ≥110 mmHg) [22]. A sensitivity analysis was then conducted based on BP values only from the 14 to 16 week visit.

The outcomes of primary interest were: PTB, SGA, PPH, and NICU admission. PTB was any birth at <37^+0^ weeks’ gestation. SGA was birthweight <10th percentile, using customized birthweight centiles adjusted for maternal weight, height, parity, ethnicity, and infant sex [23,24]. PPH was defined as an estimated blood loss ≥1,000 ml.

### Statistical analysis

Descriptive statistics summarized baseline maternal characteristics and maximal BP categories overall and according to the 2017 ACC/AHA BP categories. Differences between groups were calculated, but no *p* values were reported in accordance with STROBE guidelines.

The possible dose-response relationship between BP category and adverse outcomes was assessed in 2 ways, using available data to determine peak BP in each pregnancy. There was no imputation undertaken.

First, each BP category was treated as mutually exclusive from the others. Using the “Normal BP” category as the reference, adjusted risk ratios (aRRs) and 95% confidence intervals (CIs) were estimated using Poisson regression with robust standard errors [25]. RRs were adjusted for maternal age, body mass index (BMI), ethnicity, smoking, and alcohol use. This was an additional analysis to that in our Research Application Form (S1 Text: Application Form–SCOPE Research Project).

Second, analogous models were fitted, but the lower limit of each category was treated as a BP “cutoff” for the diagnosis of abnormal BP. In other words, for each calculation, women at or above the given BP threshold were compared with those below the threshold; for example, for “Stage 1 hypertension,” pregnancy outcomes were compared between women with sBP ≥130 mmHg or dBP ≥80 mmHg, and women with sBP <130 mmHg and dBP <80 mmHg. The diagnostic test properties were assessed using sensitivity, specificity, and positive and negative likelihood ratios (+LR and -LR, respectively). +LR was calculated as sensitivity/(1-specificity) and -LR as (1-sensitivity)/specificity. CIs were calculated by standard methods [26]. Sensitivity and specificity were considered good if >80%. LR values were interpreted as “good” if they meaningfully altered the probability of an adverse pregnancy outcome—if +LR were ≥5.0 or -LR were <0.2, according to standard criteria [27].

A sensitivity analysis was undertaken, restricting BP values to those taken in the first half of pregnancy, at 14 to 16 weeks’ gestation, and including the outcomes of preeclampsia or gestational hypertension with/without preeclampsia.

All analyses were conducted using R statistical software version 4.1.2. This study is reported as per the Strengthening the Reporting of Observational Studies in Epidemiology (STROBE) guidelines (**S1 Table**).

## Results

Of the 5,628 women recruited to SCOPE, 31 were excluded due to fetal loss at <20 weeks’ gestation (*n* = 12), termination of pregnancy due to fetal anomaly (*n* = 17), or preterm labor at <22 weeks’ gestation (*n* = 2). A total of 5,597 pregnancies were included.

The highest BP was usually in the last 2 weeks before birth (4,827, 86%), rather than at 19 to 21 weeks (428, 8%) or 14 to 16 weeks (342, 6%).

**Table 1** (gray column) shows that on average, women were in their late 20s with a BMI in the normal-overweight category. The majority were white (90.0%), with a variety of minority ethnic group women represented, particularly East Asian, South Asian, and Māori/Pacific Islanders. Using the New Zealand Socioeconomic Index (NZSEI score), 19.6% of women fell below the social deprivation threshold of 24. The most common prior medical problems were anemia and polycystic ovary syndrome, with <1% having chronic hypertension. Many women had a family history of medical problems, most commonly chronic hypertension, followed by a hypertensive disorder of pregnancy, ischemic heart disease, or type 2 diabetes mellitus.

**Table 1:** Baseline characteristics overall and according to the peak antenatal BP according to 2017 ACC-AHA BP categories* (N women (%) unless otherwise specified).

		Total	“Normal BP”	“Elevated BP”	“Stage 1 HTN”	“Stage 2 HTN”		
						All	Stage 2 (Non-severe)	Stage 2 (Severe)
Total N (Ł)		5,597 (100)	1,302 (23.3)	1,056 (18.9)	1,944 (34.7)	1,295 (23.1)	1,070 (19.1)	225 (4.0)
**Demographics**								
Maternal age^#^		28.7 (5.5)	28.9 (5.4)	28.4 (5.6)	28.7 (5.5)	28.6 (5.4)	28.7 (5.3)	28.1 (5.9)
BMI (kg/m^2^)^#^^^^		25.3 (4.9)	23.2 (3.5)	24.7 (4.4)	25.6 (4.8)	27.4 (5.8)	27.1 (5.5)	28.8 (6.8)
Ethnicity								
White		5,038 (90.0)	1,076 (82.6)	959 (90.8)	1,801 (92.6)	1,202 (92.8)	999 (93.4)	203 (90.2)
East Asian		168 (3.0)	85 (6.5)	31 (2.9)	33 (1.7)	19 (1.5)	15 (1.4)	4 (1.8)
Other		80 (1.4)	26 (2.0)	15 (1.4)	23 (1.2)	16 (1.2)	14 (1.3)	2 (0.9)
Māori/Pacific Islander		112 (2.0)	30 (2.3)	23 (2.2)	35 (1.8)	24 (1.9)	17 (1.6)	7 (3.1)
Afro-Caribbean		65 (1.2)	25 (1.9)	9 (0.9)	18 (0.9)	13 (1.0)	8 (0.7)	5 (2.2)
South Asian		134 (2.4)	60 (4.6)	19 (1.8)	34 (1.7)	21 (1.6)	17 (1.6)	4 (1.8)
Socioeconomic index (<24)		1,099 (19.6)	201 (15.4)	210 (19.9)	407 (20.9)	281 (21.7)	226 (21.1)	55 (24.4)
**Preexisting medical problems**								
Chronic hypertension (mild, no treatment)		45 (0.8)	2 (0.2)	4 (0.4)	18 (0.9)	21 (1.6)	15 (1.4)	6 (2.7)
Epilepsy		46 (0.8)	8 (0.6)	11 (1.0)	11 (0.6)	16 (1.2)	13 (1.2)	3 (1.3)
Polycystic ovarian syndrome		353 (6.3)	94 (7.2)	67 (6.3)	112 (5.8)	80 (6.2)	65 (6.1)	15 (6.7)
Anemia		738 (13.2)	179 (13.7)	143 (13.5)	269 (13.8)	147 (11.4)	128 (12.0)	19 (8.4)
Rheumatoid arthritis		31 (0.6)	4 (0.3)	5 (0.5)	13 (0.7)	9 (0.7)	8 (0.7)	1 (0.4)
**History among first degree relatives**								
Chronic hypertension		2,301 (41.1)	460 (35.3)	432 (40.9)	774 (39.8)	635 (49.0)	517 (48.3)	118 (52.4)
Type 1 diabetes mellitus		114 (2.0)	26 (2.0)	17 (1.6)	41 (2.1)	30 (2.3)	23 (2.1)	7 (3.1)
Type 2 diabetes mellitus		606 (10.8)	139 (10.7)	111 (10.5)	205 (10.5)	151 (11.7)	124 (11.6)	27 (12.0)
Ischemic heart disease		900 (16.1)	175 (13.4)	171 (16.2)	301 (15.5)	253 (19.5)	199 (18.6)	54 (24.0)
Cardiovascular accident		295 (5.3)	68 (5.2)	50 (4.7)	91 (4.7)	86 (6.6)	68 (6.4)	18 (8.0)
Any hypertensive disorder of pregnancy		1,046 (18.7)	174 (13.4)	175 (16.6)	380 (19.5)	317 (24.5)	253 (23.6)	64 (28.4)
Preeclampsia		514 (9.2)	80 (6.1)	86 (8.1)	182 (9.4)	166 (12.8)	135 (12.6)	31 (13.8)
**Current pregnancy**								
Nulliparous		ALL						
Conceived by artificial reproductive technologies		353 (6.3)	84 (6.5)	83 (7.9)	107 (5.5)	79 (6.1)	68 (6.4)	11 (4.9)
Smoking status at first SCOPE visit	No smoking	4,243 (75.8)	1,028 (79.0)	795 (75.3)	1,438 (74.0)	982 (75.8)	821 (76.7)	161 (71.6)
	Quit smoking	753 (13.5)	138 (10.6)	146 (13.8)	288 (14.8)	181 (14.0)	144 (13.5)	37 (16.4)
	Smoking	601 (10.7)	136 (10.4)	115 (10.9)	218 (11.2)	132 (10.2)	105 (9.8)	27 (12.0)
Alcohol status at first SCOPE visit	No alcohol	2,173 (38.8)	516 (39.6)	458 (43.4)	691 (35.5)	508 (39.2)	406 (37.9)	102 (45.3)
	Quit drinking	2,855 (51.0)	660 (50.7)	504 (47.7)	1,030 (53.0)	661 (51.0)	563 (52.6)	98 (43.6)
	Continued to drink	569 (10.2)	126 (9.7)	94 (8.9)	223 (11.5)	126 (9.7)	101 (9.4)	25 (11.1)
Multivitamin at first SCOPE visit	No	2,760 (49.3)	644 (49.5)	499 (47.3)	955 (49.1)	662 (51.1)	546 (51.0)	116 (51.6)
	Yes	2,820 (50.4)	654 (50.2)	553 (52.4)	980 (50.4)	633 (48.9)	524 (49.0)	109 (48.4)
Folate at first SCOPE visit	No	1,697 (30.3)	387 (29.7)	316 (29.9)	599 (30.8)	395 (30.5)	322 (30.1)	73 (32.4)
	Yes	3,900 (69.7)	915 (70.3)	740 (70.1)	1,345 (69.2)	900 (69.5)	748 (69.9)	152 (67.6)

* The highest antenatal BP was determined from values at the 3 time points available (i.e., 14–16 weeks, 19–21 weeks, and during the 2 weeks before birth), as part of the original SCOPE data collection. BP was categorized according to the 2017 ACC/AHA criteria as follows: normal (sBP <120 mmHg and dBP <80 mmHg), elevated (sBP 120–129 mmHg but dBP <80 mmHg), stage 1 hypertension (sBP 130–139 mmHg or dBP 80–89 mmHg), and stage 2 hypertension (sBP ≥140 mmHg or dBP ≥ 90mmHg).

Ł Where women are missing from data points, percentages are calculated from total number of women with data available.

# Mean (standard deviation).

^ BMI recorded at booking visit.

ACC, American College of Cardiology; AHA, American Heart Association; BMI, body mass index; BP, blood pressure; dBP, diastolic blood pressure; HTN, hypertension; sBP, systolic blood pressure.

In the index pregnancy, all women were nulliparous (according to study inclusion criteria), with few conceiving based on artificial reproductive technologies (**Table 1**, gray column). Most were non-smokers or quit when pregnant and abstained from alcohol once pregnant. At the first SCOPE visit (at 14 to 16 weeks’ gestation), half were taking a multivitamin and two-thirds folic acid specifically. Overall, 23.3% of women remained normotensive throughout the duration of their pregnancy, 18.9% were classified as having “Elevated BP,” 34.7% as “Stage-1 hypertension,” and 23.1% as “Stage-2 hypertension” (19.1% non-severe and 4% severe) (**Table 1**, white columns).

Across BP categories, baseline characteristics differed (**Table 1**, **white columns**). Women in higher BP categories more often had higher BMI, were white and had NZSEI scores consistent with social deprivation. With increasing BP category, more women had a personal or family history of chronic hypertension or a family history of hypertensive pregnancy or ischemic heart disease.

**Table 2** (gray column) shows that on average, women were just under 40 weeks’ gestation at birth, with one-third undergoing labor induction. Over one-quarter of women delivered by operative vaginal delivery and just over one-quarter by cesarean. There was 1 maternal death. Preeclampsia developed in 5.0% and gestational hypertension in 8.4% of women overall.

**Table 2:** Labor, delivery, and maternal and perinatal outcomes overall and according to peak antenatal BP according to 2017 ACC/AHA BP categories* (mean ± SD or median [IQR] N (%) unless otherwise specified).

	Total	“Normal BP”	“Elevated BP”	“Stage 1 HTN”	“Stage 2 HTN”		
					All	“Stage 2” (Non-severe)	“Stage 2” (Severe)
Total N (Ł)	5,597	1,302 (23.3)	1,056 (18.9)	1,944 (34.7)	1,295 (23.1)	1,070 (19.1)	225 (4.0)
**Labor and delivery outcomes**							
GA delivery (weeks)	39.7 (2.0)	39.6 (2.0)	39.7 (2.0)	39.9 (1.8)	39.3 (2.3)	39.6 (2.1)	38.0 (2.9)
Induction or prelabor rupture of membranes + augmentation	1,830 (32.7)	336 (25.8)	292 (27.7)	617 (31.7)	585 (45.2)	454 (42.4)	131 (58.2)
*(Missing)*	*83 (1*.*5)*	*32 (2*.*5)*	*16 (1*.*5)*	*18 (0*.*9)*	*17 (1*.*3)*	*17 (1*.*6)*	*0 (0*.*0)*
Mode of birth							
Unassisted vaginal	2,521 (45.0)	629 (48.3)	501 (47.4)	905 (46.6)	486 (37.5)	413 (38.6)	73 (32.4)
Operative vaginal	1,482 (26.5)	354 (27.2)	277 (26.2)	514 (26.4)	337 (26.0)	293 (27.4)	44 (19.6)
Prelabor cesarean	498 (8.9)	106 (8.1)	78 (7.4)	135 (6.9)	179 (13.8)	124 (11.6)	55 (24.4)
Cesarean (in labor)	1,093 (19.5)	211 (16.2)	200 (18.9)	389 (20.0)	293 (22.6)	240 (22.4)	53 (23.6)
Maternal death antepartum	1 (0.0)	0 (0.0)	0 (0.0)	1 (0.1)	0 (0.0)	0 (0.0)	0 (0.0)
*(Missing)*	*2 (0*.*0)*	*2 (0*.*2)*	*0 (0*.*0)*	*0 (0*.*0)*	*0 (0*.*0)*	*0 (0*.*0)*	*0 (0*.*0)*
**Maternal morbidity**							
Type of HDP							
Chronic hypertension Missing	17 (0.3)	0 (0.0)	0 (0.0)	1 (0.1)	16 (1.2)	8 (0.7)	8 (3.6)
	24 (0.4)	0 (0.0)	0 (0.0)	0 (0.0)	24 (1.9)	16 (1.5)	8 (3.6)
Preeclampsia Missing	278 (5.0)	1 (0.1)	0 (0.0)	6 (0.3)	271 (20.9)	143 (13.4)	128 (56.9)
	*5 (0*.*1)*	*5 (0*.*4)*	*0 (0*.*0)*	*0 (0*.*0)*	*0 (0*.*0)*	*0 (0*.*0)*	*0 (0*.*0)*
Gestational hypertension	470 (8.4)	0 (0.0)	0 (0.0)	4 (0.2)	466 (36.0)	382 (35.7)	84 (37.3)
**Serious maternal complications**							
Placental abruption	41 (0.7)	8 (0.6)	7 (0.7)	11 (0.6)	15 (1.2)	9 (0.8)	6 (2.7)
Major postpartum hemorrhage *(Missing)*	227 (4.1)	45 (3.5)	42 (4.0)	71 (3.7)	69 (5.3)	62 (5.8)	7 (3.1)
	*909 (16*.*2)*	*182 (14*.*0)*	*115 (10*.*9)*	*365 (18*.*8)*	*247 (19*.*1)*	*215 (20*.*1)*	*32 (14*.*2)*
**Fetal and neonatal outcomes**							
Fetal outcome							
Intrauterine fetal death/stillbirth	24 (0.4)	6 (0.5)	5 (0.5)	8 (0.4)	5 (0.4)	3 (0.3)	2 (0.9)
Liveborn	5,566 (99.4)	1,294 (99.4)	1,050 (99.4)	1,935 (99.5)	1,287 (99.4)	1,065 (99.5)	222 (98.7)
Neonatal death	7 (0.1)	2 (0.2)	1 (0.1)	1 (0.1)	3 (0.2)	2 (0.2)	1 (0.4)
Preterm birth							
<34 weeks	108 (1.9)	24 (1.8)	16 (1.5)	23 (1.2)	45 (3.5)	20 (1.9)	25 (11.1)
<37 weeks (total)	342 (6.1)	69 (5.3)	54 (5.1)	94 (4.8)	125 (9.7)	69 (6.4)	56 (24.9)
<37 weeks (spontaneous)	231 (4.1)	58 (4.5)	48 (4.5)	79 (4.1)	46 (3.6)	41 (3.8)	5 (2.2)
Sex							
Male	2,840 (50.7)	662 (50.8)	514 (48.7)	1,016 (52.3)	648 (50.0)	533 (49.8)	115 (51.1)
Female	2,757 (49.3)	640 (49.2)	542 (51.3)	928 (47.7)	647 (50.0)	537 (50.2)	110 (48.9)
Birthweight							
Small for gestational age	624 (11.1)	115 (8.8)	94 (8.9)	188 (9.7)	227 (17.5)	172 (16.1)	55 (24.4)
Large for gestational age *Missing*	531 (9.5)	130 (10.0)	118 (11.2)	170 (8.7)	113 (8.7)	92 (8.6)	21 (9.3)
	*3 (0*.*1)*	*1 (0*.*1)*	*2 (0*.*2)*	*0 (0*.*0)*	*0 (0*.*0)*	*0 (0*.*0)*	*0 (0*.*0)*
Low Apgar score (<7 at 5 min) *Missing*	61 (1.1)	12 (0.9)	12 (1.1)	15 (0.8)	22 (1.7)	16 (1.5)	6 (2.7)
	*60 (1*.*1)*	*29 (2*.*2)*	*9 (0*.*9)*	*14 (0*.*7)*	*8 (0*.*6)*	*6 (0*.*6)*	*2 (0*.*9)*
Intubation at birth							
Yes	49 (0.9)	6 (0.5)	11 (1.0)	11 (0.6)	21 (1.6)	15 (1.4)	6 (2.7)
*(Missing)*	*29 (0*.*5)*	*14 (1*.*1)*	*0 (0*.*0)*	*7 (0*.*4)*	*8 (0*.*6)*	*7 (0*.*7)*	*1 (0*.*4)*
Admission to NICU	647 (11.6)	116 (8.9)	102 (9.7)	190 (9.8)	239 (18.5)	165 (15.4)	74 (32.9)

Stillbirth defined as fetal death in utero ≥20+0 weeks gestation.

* The highest antenatal BP was determined from values at the 3 time points available (i.e., 14–16 weeks, 19–21 weeks, and during the 2 weeks before birth), as part of the original SCOPE data collection. BP was categorized according to the 2017 ACC/AHA criteria as follows: “Normal” (sBP <120 mmHg and dBP <80 mmHg), “Elevated BP” (sBP 120–129 mmHg but dBP <80 mmHg), “Stage 1 hypertension” (sBP 130–139 mmHg and/or dBP 80–89 mmHg), and “Stage 2 hypertension” (sBP ≥140 mmHg and/or dBP ≥90 mmHg).

Ł Where women are missing from data points, percentages are calculated from total number of women with data available.

ACC, American College of Cardiology; AHA, American Heart Association; BP, blood pressure; dBP, diastolic blood pressure; HTN, hypertension; NA, not applicable; NICU, neonatal intensive care unit; sBP, systolic blood pressure.

Across BP categories, pregnancy outcomes differed (**Table 2**, white columns). Women with higher BP more often: delivered earlier, had higher rates of induction of labor and cesarean birth (pre-labor and in-labor); had a history of chronic hypertension not requiring antihypertensive therapy, preeclampsia, or gestational hypertension; experienced placental abruption (but not PPH); delivered at under 34 or 37 weeks’ gestation; and had an SGA infant with low Apgars (<7) at 5 min who required admission to the NICU. Given that hypertensive disorders of pregnancy require a BP in the “Stage 2 hypertension” category, these diagnoses were seen mainly in the “Stage 2 hypertension” group.

**Table 3** shows that there was an association between “Stage 2 hypertension” (particularly severe “Stage 2 hypertension”) and PTB, SGA, and NICU admission. The association between BP categories and the risk of PPH was unclear. Unadjusted and adjusted analyses showed similar results.

**Table 3:** Adjusted risk ratios and 95% CIs for adverse pregnancy outcomes, according to peak antenatal BP according to 2017 ACC-AHA BP categories (mmHg)*.

	Antenatal BP (mmHg)ł				
	Normal	“Elevated BP”	“Stage 1 HTN”	“Stage 2 HTN” (Non-severe)	“Stage 2 HTN” (Severe)
	<120/<80	120–129/<80	130–139/80–89	140–159/90–109	≥160/≥110
	(*N* = 1,302)	(*N* = 1,056)	(*N* = 1,944)	(*N* = 1,070)	(*N* = 225)
**PPH >1L**					
Event rate (n/N)	45/1,120	42/941	71/1,579	62/855	7/193
RR [95% CI]	1.00 (Ref)	1.11 (0.74, 1.68)	1.12 (0.78, 1.61)	1.80 (1.24, 2.62)	0.90 (0.41, 1.97)
aRR [95% CI]	1.00 Ref	1.09 (0.72, 1.65)	1.04 (0.72, 1.50)	1.58 (1.07, 2.33)	0.72 (0.32, 1.62)
**Preterm birth**					
Event rate (n/N)	69/1,302	54/1,056	94/1,944	69/1,070	54/225
RR [95% CI]	1.00 (Ref)	0.96 (0.68, 1.36)	0.91 (0.67, 1.23)	1.22 (0.88, 1.68)	**4.70 (3.40, 6.49)**
aRR [95% CI]	1.00 (Ref)	0.97 (0.68, 1.37)	0.94 (0.69, 1.27)	1.26 (0.90, 1.76)	**4.88 (3.46, 6.88)**
**Birthweight <10th centile**					
Event rate (n/N)	115/1,302	94/1,056	188/1,944	172/1,070	55/225
RR [95% CI]	1.00 (Ref)	1.01 (0.78, 1.31)	1.09 (0.88, 1.37)	**1.82 (1.46, 2.27)**	**2.77 (2.07, 3.69)**
aRR [95% CI]	1.00 (Ref)	1.02 (0.79, 1.33)	1.09 (0.87, 1.37)	**1.82 (1.45, 2.29)**	**2.70 (2.00, 3.65)**
**Neonatal unit admission**					
Event rate (n/N)	116/1,302	102/1,056	190/1,944	165/1,070	74/225
RR [95% CI]	1.00 (Ref)	1.08 (0.84, 1.40)	1.10 (0.88, 1.37)	**1.73 (1.38, 2.16)**	**3.69 (2.86, 4.76)**
aRR [95% CI]	1.00 (Ref)	1.05 (0.81, 1.35)	1.06 (0.84, 1.32)	**1.65 (1.31, 2.07)**	**3.4 (2.59, 4.46)**

* The highest antenatal BP was determined from values at the 3 time points available (i.e., 14–16 weeks, 19–21 weeks, and during the 2 weeks before birth), as part of the original SCOPE data collection. BP was categorized according to the 2017 ACC/AHA criteria as follows: “Normal” (sBP <120 mmHg and dBP <80 mmHg), “Elevated BP” (sBP 120–129 mmHg but dBP <80 mmHg), “Stage 1 hypertension” (sBP 130–139 mmHg and/or dBP 80–89 mmHg), and “Stage 2 hypertension” (sBP ≥140 mmHg and/or dBP ≥90 mmHg). Risk ratios and 95% CIs were estimated using Poisson regression with robust standard errors, using “Normal” BP as the reference category. RRs were adjusted for maternal age, BMI at booking, ethnicity, smoking status, and alcohol use.

ACC, American College of Cardiology; AHA, American Heart Association; aRR, adjusted risk ratio; BMI, body mass index; BP, blood pressure; CI, confidence interval; dBP, diastolic blood pressure; HTN, hypertension; PPH, postpartum hemorrhage; sBP, systolic blood pressure.

**Table 4** shows the diagnostic test properties of each BP category as a threshold for an abnormal BP. Only “Elevated BP” had a sensitivity of 80% or more for any of the outcomes but specificity was low (<25%). Specificity was at least 80% only for severe “Stage 2 hypertension,” for all outcomes examined. The LR+ was considered good only for “Stage 2 hypertension” and PTB; all other upper 95% CI of the +LR were <5.0 for other outcomes. No LR- point estimate or lower 95% CI was good enough (i.e., <0.20) to reassure that adverse outcomes were unlikely.

**Table 4:** Sensitivity, specificity, and likelihood ratios for SCOPE outcomes according to peak antenatal BP according to BP thresholds set by the 2017 ACC/AHA BP categories*.

	Antenatal BP (mmHg)*				
	Normal	“Elevated BP”	“Stage 1 HTN”	“Stage 2 HTN” (Non-severe)	“Stage 2 HTN” (Severe)
	<120/<80	120–129/<80	130–139/80–89	140–159/90–109	≥160/≥110
	(*N* = 1,302)	(*N* = 1,056)	(*N* = 1,944)	(*N* = 1,070)	(*N* = 225)
**PPH >1L**					
Event rate (n/N)	227/4,688	182/3,571	140/2,627	69/1,048	7/193
Sensitivity [95% CI]	*Ref*	0.80 (0.74, 0.85)	0.62 (0.55, 0.68)	0.30 (0.24, 0.37)	0.03 (0.01, 0.06)
Specificity [95% CI]	*Ref*	0.24 (0.23, 0.25)	0.44 (0.43, 0.46)	0.78 (0.77, 0.79)	0.96 (0.95, 0.96)
LR positive [95% CI]ł	*Ref*	1.06 (0.99, 1.13]	1.11 (1.00, 1.23)	1.39 (1.13, 1.70)	0.74 (0.35, 1.55)
LR negative [95% CI]ł	*Ref*	0.82 (0.63, 1.07]	0.87 (0.73, 1.02)	0.89 (0.82, 0.97)	1.01 (0.99, 1.04)
**Preterm birth**					
Event rate (n/N)	342/5,597	273/4,295	219/3,239	125/1,295	56/255
Sensitivity [95% CI]	*Ref*	0.80 (0.75, 0.84)	0.64 (0.59, 0.69)	0.37 (0.31, 0.42)	0.16 (0.13, 0.21)
Specificity [95% CI]	*Ref*	0.23 (0.22, 0.25)	0.43 (0.41, 0.44)	0.78 (0.77, 0.79)	0.97 (0.96, 0.97)
LR positive [95% CI]ł	*Ref*	1.06 (1.02, 1.11)	1.11 (1.03, 1.21)	1.64 (1.42, 1.90)	5.09 (3.84, 6.75)
LR negative [95% CI]ł	*Ref*	0.80 (0.68, 0.95)	0.85 (0.73, 0.98)	0.82 (0.75, 0.89)	0.86 (0.82, 0.91)
**Birthweight <10th percentile**					
Event rate (n/N)		509/4,295	415/3,239	227/1,295	55/225
Sensitivity [95% CI]	*Ref*	0.82 (0.78, 0.85)	0.67 (0.63, 0.70)	0.36 (0.33, 0.40)	0.09 (0.07, 0.11)
Specificity [95% CI]	*Ref*	0.24 (0.23, 0.25)	0.43 (0.42, 0.45)	0.79 (0.77, 0.80)	0.97 (0.96, 0.97)
LR positive [95% CI]ł	*Ref*	1.07 (1.03, 1.12)	1.17 (1.10, 1.24)	1.69 (1.51, 1.90)	2.58 (1.92, 3.45)
LR negative [95% CI]ł	*Ref*	0.77(0.65, 0.92)	0.78 (0.69, 0.87)	0.81 (0.76, 0.86)	0.94 (0.92, 0.97)
**Neonatal unit admission**					
Event rate (n/N)		531/4,295	429/3,239	239/1,295	74/225
Sensitivity [95% CI]	*Ref*	0.82 (0.79, 0.85)	0.66 (0.63, 0.70)	0.37 (0.33, 0.41)	0.11 (0.09, 0.14)
Specificity [95% CI]	*Ref*	0.24 (0.23, 0.25)	0.43 (0.42, 0.45)	0.79 (0.77, 0.80)	0.97 (0.96, 0.97)
LR positive [95% CI]ł	*Ref*	1.08 (1.04, 1.12)	1.17 (1.10, 1.24)	1.73 (1.54, 1.94)	3.75 (2.87, 4.89)
LR negative [95% CI]ł	*Ref*	0.75 (0.63, 0.89)	0.78 (0.70, 0.87)	0.80 (0.75, 0.85)	0.91 (0.89, 0.94)

* As assessed at outpatient antenatal visits or medical assessment unit visits prior to the delivery admission. BP was categorized according to the 2017 ACC/AHA criteria as follows: normal (sBP <120 mmHg and dBP <80 mmHg), elevated (sBP 120–129 mmHg but dBP <80 mmHg), Stage 1 hypertension (sBP 130–139 mmHg and/or dBP 80–89 mmHg), and Stage 2 hypertension (sBP ≥140 mmHg and/or dBP ≥90 mmHg). Diagnostic test properties were calculated for the lower limit of each category and above, vs. BP values below the category. +LR was calculated as sensitivity/(1-specificity) and -LR as (1-sensitivity)/specificity.

ł Likelihood ratios were calculated for each category and above, vs. the category(ies) below.

ACC, American College of Cardiology; AHA, American Heart Association; BP, blood pressure; CI, confidence interval; FP, false positive; HTN, hypertension; LR, likelihood ratio; PPH, postpartum hemorrhage.

When restricted to BP measurements taken at 14 to 16 weeks, a similar pattern of association was seen (**S2 Table**), but with very poor diagnostic test performance for any BP category (**S3 Table**). Preeclampsia and gestational hypertension with/without preeclampsia were significantly more likely in women with “Stage 2 hypertension” at this early gestation.

## Discussion

In a cohort of standard-risk nulliparous women, using 2017 ACC/AHA BP thresholds below the traditional 140/90 mmHg (“Stage 2 hypertension”) in pregnancy, an additional 35% of women would have been classified as having an abnormal BP. Use of “Elevated BP” or “Stage 1 hypertension,” below the current “Stage 2 hypertension” threshold of 140 mmHg systolic and/or 90 mmHg diastolic, would not have identified an increased risk of PPH, PTB, SGA infants, or NICU admission, and importantly, would not have identified women at increased risk of adverse pregnancy outcomes. While “Stage-2 hypertension” was associated with an increased risk of PTB, SGA, and NICU admission, only for severe “Stage 2 hypertension” would this have usefully identified women at risk of PTB who should already be on a higher risk care pathway. There was no BP level that could usefully reassure about development of any of the outcomes examined.

In most contexts, women would have been labeled as “high risk” if identified as having hypertension, and this would limit her choice of care pathway in the index pregnancy. Guidelines mandate additional enhanced surveillance of maternal and fetal well-being, including consideration of timed birth [28,29]. Also, given potential recurrence of the hypertensive disorders of pregnancy, the care pathway may be altered in subsequent pregnancies, including recommendations for preventative therapy [28]. This can be justified only if there are benefits to being labeled high-risk, and our findings suggest that this is not the case.

Since the publication of the ACC/AHA categories in 2017, there have been many reports of the application of these categories in pregnancy and their relationship with adverse pregnancy outcomes. Most publications have focused on BP values at <20 weeks’ gestation [12,14,16,17,30–35]. Recent systematic reviews have identified that there are associations between adverse pregnancy outcomes and each of “Elevated BP” and “Stage 1 hypertension”; however, based on LRs, the corresponding BP thresholds (i.e., 120 mmHg systolic or 130/80 mmHg, respectively) perform poorly in identifying women at either increased or decreased risk of adverse outcomes [36–38]. That work led to conclusions that, to the best of our knowledge, there is no justification for lowering the BP threshold for normality in pregnancy, given the increased proportion of women who would then be cared for on high-risk care pathways, without a demonstrable reduction in adverse pregnancy outcomes.

Only 3 studies included in our prior systematic reviews [13,18,39] of the relationship between 2017 ACC/AHA BP categories have considered nulliparous women alone, with 2 [13,18] investigating BP values in the second half of pregnancy. Risk assessment in nulliparous women cannot, by definition, include obstetric history to guide care. As the maximal BP in the present study was in the 2 weeks before birth, our results are most compatible with the finding from 33 to 36 weeks’ gestation subgroups of relevant studies of women with heterogeneous parity [40]. In that subgroup (unlike our findings), “Elevated BP” and “Stage 1 hypertension” were associated with a higher risk of PTB and NICU admission; however, in common with the present study, “Stage 2 hypertension” was associated with more PTB, SGA infants, and NICU admission. Furthermore, in our study, neither “Elevated BP,” “Stage 1 hypertension,” nor “Stage 2 hypertension” could usefully identify nulliparous women at increased or decreased risk of any of these outcomes. Unlike our study, PPH was not an outcome reported in the systematic review.

The SCOPE cohort was a large, prospective, international cohort of nulliparous women. BP measurements were taken by trained study nurses using a validated device and a standardized method, increasing the reliability of these results. To the best of our knowledge, our analysis is the first to compare use of the 2017 ACC/AHA BP categories both in this specific population of women and as diagnostic criteria.

A limitation of our analysis is the ethnic homogeneity of the SCOPE data, which does not reflect the ethnic heterogeneity of the communities in which the data were collected and limits the generalizability of our study findings. The BP values recorded at the routine SCOPE visits at 14 to 16 and 19 to 21 weeks were standardized and collected according to the study protocol. The information collected on BPs within 2 weeks before birth were collected from clinical notes; therefore, it cannot be confirmed that the same rigor was used for recording these measurements. The late pregnancy BPs were the peak antenatal BPs for the majority of women; hence, the majority of women were categorized according to measurements taken from the clinical record. Although this probably improves the external validity of the study findings, it may have led to misclassification of some women. While our analysis used BP values recorded within 2 weeks before delivery, BP could have risen further [38], so it is possible that the BP levels at or after birth were higher; however, there were only 7 women (0.2%) who developed intrapartum or postpartum preeclampsia who had BP values below 140/90 mmHg throughout their pregnancy. The original SCOPE study asked all women if they were taking aspirin at their initial antenatal visit, but there were no further re-checks during ongoing pregnancy; however, it is likely that few women, if any, were taking aspirin, as participants were regarded as low-risk for preeclampsia and SCOPE recruitment antedated evidence of aspirin prevention for PTB [41]. Finally, we were lacking further detail about some outcomes, including whether PTB was iatrogenic or spontaneous; this outcome occurred in a minority of women.

The current analyses in a nulliparous cohort reinforce the importance of continued close clinical surveillance in late pregnancy, as no BP level provided reassurance against the development of the adverse pregnancy outcomes examined. Use of BP as a continuous variable in prediction models (such as for preeclampsia [42]) may identify increased risk (such as of preeclampsia) at BP levels below 140/90 mmHg, and aspects of BP other than the level (e.g., variability of BP level between visits) may further aid in identifying women at risk of adverse pregnancy outcomes, who could be recruited to intervention (prevention) trials, such as with aspirin [43]. To cost-effectively optimize outcomes, when defining diagnostic thresholds, the increased resource utilization required for surveillance of women labeled as having pregnancy hypertension needs to be considered carefully [44]; this is especially true in resource-constrained low- and middle-income countries which carry the largest burden of HDP-related adverse outcomes [45].

## Conclusion

Our findings suggest that among nulliparous women, as in the general maternity population, the 2017 ACC/AHA BP categories are associated with adverse pregnancy outcomes, but they are unable to usefully distinguish between pregnancies at risk of those adverse outcomes, and those that are not. This study does not support implementation in pregnancy of the lower, 2017 ACC/AHA BP thresholds among nulliparous women in pregnancy.

## Supporting information

S1 Fig Derivation of the SCOPE cohort. (DOCX)

S1 Text Application Form–SCOPE Research Project. (DOC)

S1 Table Adjusted risk ratios for adverse pregnancy outcomes, according to the 2017 ACC-AHA BP categories (mmHg) based on BPs at 14 to 16 weeks. (DOCX)

S2 Table Adjusted risk ratios for adverse pregnancy outcomes, according to the 2017 ACC-AHA BP categories (mmHg) based on BPs at 14 to 16 weeks. (DOCX)

S3 Table Sensitivity, specificity, and likelihood ratios for SCOPE outcomes according to BP thresholds set by the 2017 ACC/AHA BP categories applied to BPs at 14 to 16 weeks gestation. (DOCX)

## Abbreviations

ACC: American College of Cardiology
ACOG: American College of Obstetricians and Gynecologists
AHA: American Heart Association
aRR: adjusted risk ratio
BMI: body mass index
BP: blood pressure
CI: confidence interval
dBP: diastolic blood pressure
HDP: hypertensive disorder of pregnancy
LR: likelihood ratio
NICU: neonatal intensive care unit
NZSEI: New Zealand Socioeconomic Index
PPH: postpartum hemorrhage
PTB: preterm birth
sBP: systolic blood pressure
SGA: small-for-gestational age
