# Supplementary material for: Diagnostic properties of differing BP thresholds for adverse pregnancy outcomes in standard-risk nulliparous women: A secondary analysis of SCOPE cohort data
Source: PLoS Med. 2025 Jan 22;22(1):e1004471. doi: 10.1371/journal.pmed.1004471 (PMC11798451; doi:10.1371/journal.pmed.1004471)
Supplement: S1 Fig — (DOCX) [file pmed.1004471.s001.docx]

**S1 Fig** – Derivation of the SCOPE cohort


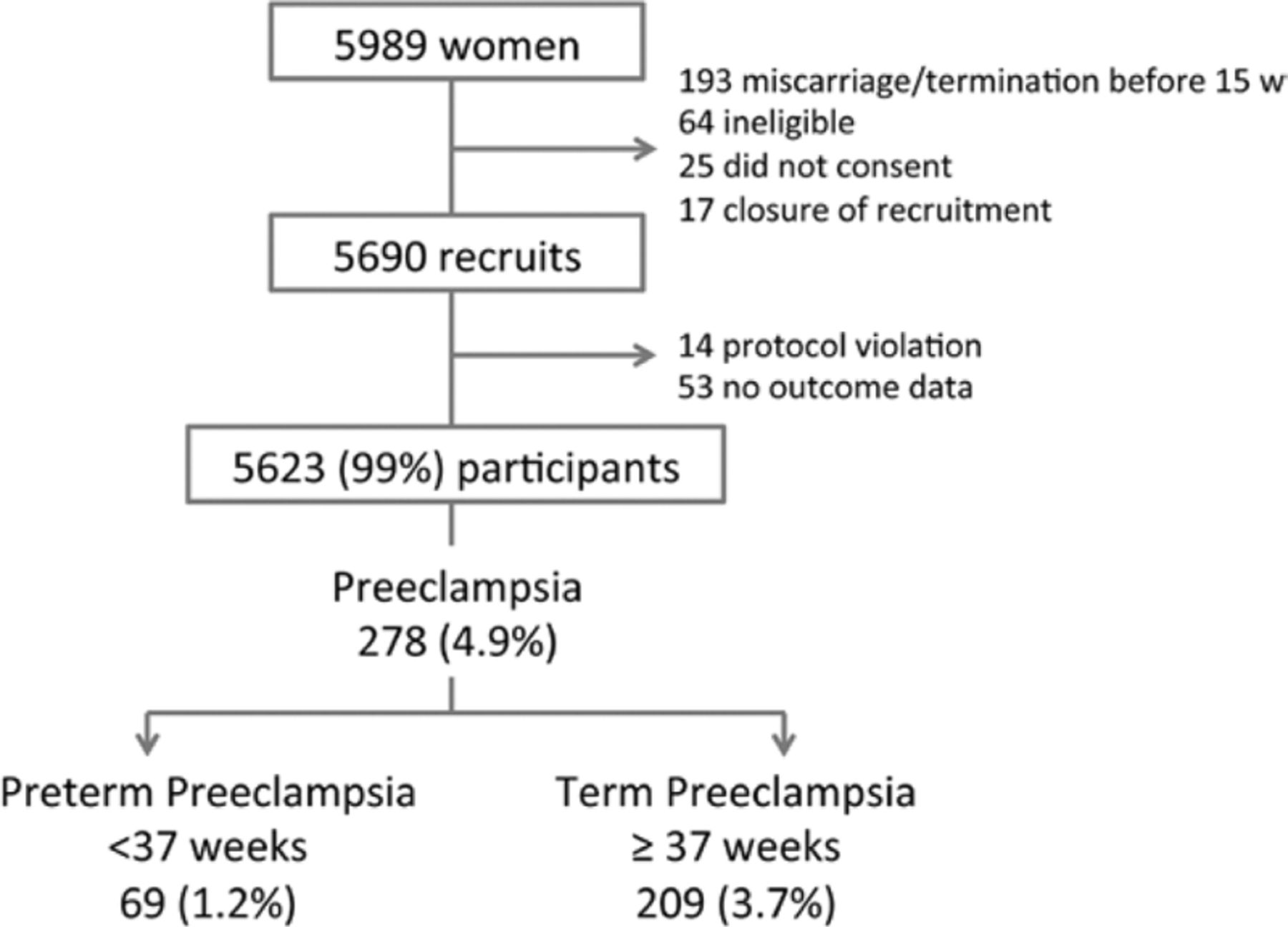


Ref: Kenny LC, Black MA, Poston L, Taylor R, Myers JE, Baker PN, McCowan LM, Simpson NA, Dekker GA, Roberts CT, Rodems K, Noland B, Raymundo M, Walker JJ, North RA. Early pregnancy prediction of preeclampsia in nulliparous women, combining clinical risk and biomarkers: the Screening for Pregnancy Endpoints (SCOPE) international cohort study. Hypertension. 2014 Sep;64(3):644-52. doi: 10.1161/HYPERTENSIONAHA.114.03578. PMID: 25122928.
