# Supplementary material for: Diagnostic properties of differing BP thresholds for adverse pregnancy outcomes in standard-risk nulliparous women: A secondary analysis of SCOPE cohort data
Source: PLoS Med. 2025 Jan 22;22(1):e1004471. doi: 10.1371/journal.pmed.1004471 (PMC11798451; doi:10.1371/journal.pmed.1004471)
Supplement: S1 Text — (DOC) [file pmed.1004471.s002.doc]

**APPLICATION FORM**

**SCOPE RESEARCH PROJECT**

**RAF Number: 4.38**

**Project Title (Short please):** Do lower blood pressure (BP) cut-offs in pregnancy identify women at greater risk of adverse maternal and perinatal outcome?

**Project PI:** Laura A. Magee

**Other Researcher(s):** Peter von Dadelszen, Asma Khalil, , Jeff Bone, Paul Seed (to be confirmed), Fergus McCarthy, Jenny Myers, Jimmy Walker, Dharmintra Pasupathy, Lucilla Poston, Gus Dekker, Claire Roberts, Lesley McCowan.

**Date of Submission: 3 November 2020**

**Mark X** in each centre involved in the research

|  | SCOPE Centres involved in research | SCOPE Biobanks Data involved in research | SCOPE Biobanks Specimen use in research |
| --- | --- | --- | --- |
| Auckland | X |  |  |
| Adelaide | X |  |  |
| Manchester | X |  |  |
| Kings | X |  |  |
| Leeds | X |  |  |
| Cork | X |  |  |

**Funding required to do project yes no** (delete as appropriate)

| **Funding Status** | **Yes** | **No** | **N.A.** | **Funder/Amount funded/Date** |
| --- | --- | --- | --- | --- |
| Applied for funding |  | X |  |  |
| Planning to apply for funding | X |  |  |  |
| Funder/Potential Funder |  |  |  | Research for Patient Benefit (RfPB, 2021) |
| Date expect to hear outcome of funding application |  |  |  |  |
| Date heard outcome of application |  |  | X |  |
| Funded |  |  | X |  |
| Amount funded |  |  | X |  |

**Ethics**

Ethical Approval is required for all SCOPE clinical centres, and women consent prior to participating. Most projects should be covered by this consent, but there maybe occasion when additional ethical approval is required.

**Ethics Approval complete**  No (but will make an application if advised that the SCOPE ethical approval does not cover the project).

**Collaboration with Commercial Institution or Academic Inst. outside SCOPE Consortium outside**

No (not for this project)

**If yes, please complete this table:**

| **Commercial Details** | **Complete details** |
| --- | --- |
| Name of Company/ Academic Institution |  |
| Type of Collaboration | - fee for service contract, no IP issues - collaboration in kind, with IP sharing |
| IP ownership solved | yes / no |
| If IP ownership solved, please summarise solution |  |
| If IP ownership not solved, summarise issues |  |

Note: If more than 1 institution involved please copy the rows in table and complete for each institution

**Research Proposal (max 1 page)**

**Summary Abstract (1-2 paragraphs)**

Measuring blood pressure in pregnancy is a key part of antenatal care, because high blood pressure is associated with an increased risk of problems for mothers (such as bleeding) and babies (such as poorer growth). High blood pressure in pregnancy is diagnosed when the systolic (top number) of the blood pressure reading reaches 140 or more, or the bottom number reaches 90 or more (that is, 140/90 or above). However, it has been suggested that use of a cut-off of 130/80 or even just systolic number of 120-129 would identify more mothers and babies at risk. We wish to study whether this is indeed the case.

**Aims/Hypotheses**:

To compare the risk of adverse pregnancy outcomes according to BP level, as a continuous variable and according to the American Heart Association (AHA)/American College of Cardiology (ACC) criteria: normal (BP <120/80mmHg), pre-hypertension (120-129/<80mmHg), stage 1 hypertension (BP 130-139/80-89mmHg), and stage 2 hypertension (BP ≥140/90mmHg).

**Background: (1 paragraph):** Clinical practice guidelines define hypertension as a systolic blood pressure (BP) ≥140mmHg or a diastolic BP ≥90mmHg(1). However, there is a continuous relationship between higher BP and worse maternal outcomes among women with any type of pregnancy hypertension, as well as those with pre-eclampsia specifically(2-5). Severe elevations of BP (to ≥160/110 mm Hg, regardless of the hypertensive disorder) are also associated with adverse maternal and perinatal outcomes(6). While a cut-off of 140/90mmHg is consistent with how hypertension is generally defined outside pregnancy, the American College of Cardiology/American Heart Association (ACC/AHA) have lowered their threshold for diagnosis to 130/80mmHg, with 130-139/80-89mmHg designated as stage 1 hypertension and ≥140/90mmHg as stage 2(7). The American College of Obstetricians and Gynecologists (ACOG) has not yet adopted a 130/80mmHg threshold for hypertension in pregnancy, however, there is a literature emerging on the relationship between a threshold of 130/80mmHg for diagnosing hypertension in pregnancy and adverse outcomes. Evidence suggests that more women would be identified who have a heightened risk of pre-eclampsia, preterm birth, and gestational diabetes, with the risk being intermediate between those with BP <130/80 mmHg (defined as normal if <120/80mmHg and ‘elevated’ if systolic is 120-129) and stage 2 chronic hypertension (with BP ≥140/90mmHg)(8, 9). Furthermore, in addition to being at increased risk of pre-eclampsia, it would appear that women with stage 1 hypertension (i.e., BP of 130-139/80-89mmHg) would benefit from low-dose aspirin for pre-eclampsia prevention, based on a secondary analysis of trial data(10).

**Methods**

Study Design: Prospective cohort study of pregnancy outcome according to highest antenatal BP value: normal (<120/80mmHg), pre-hypertension (120-129/<80mmHg), stage 1 hypertension (130-139/80-89mmHg), and stage 2 hypertension (≥140/90mmHg, diagnosis of chronic hypertension, or treatment with antihypertensive therapy).

Baseline maternal and pregnancy characteristics of interest include: demographics, past history, and current pregnancy. Antenatal surveillance of maternal and fetal well-being include: N and type of antenatal care outpatient visits, inpatient stays (and duration), N tests of fetal well-being (including outpatient cardiotocograms and inpatient/outpatient ultrasound scans). Maternal and perinatal complications of pregnancy include: pre-eclampsia, gestational diabetes mellitus, induction, mode of delivery, preterm birth, stillbirth, neonatal death, birthweight <10th or >90th centiles, neonatal care unit admission (and duration and stay >=4 hr or >=48hr).

We will compare the sensitivity and specificity of antenatal BP readings for detection of the adverse pregnancy outcomes in the Methods, above.

Type(s) and Numbers of Participants: 5628 women in SCOPE cohort who have data on antenatal BP and pregnancy outcomes.

Type(s) and volume of specimens required: None.

*All blood specimens 250ul aliquots, urine 0.9ml aliquot

**Other Comments**: This will inform the definition of pregnancy hypertension.

**Planned commencement date:** 01 Jan 2021

**Expected finish date:** 31 Jul 2021

**Project Agreement required** No

**Project Agreement completed** NA **yet to be completed** NA

**References:**

1. Scott G, Gillon TE, Pels A, von Dadelszen P, Magee LA. Guidelines - similarities/dissimilarities. A systematic review of international clinical practice guidelines for pregnancy hypertension. Am J Obstet Gynecol. 2020;[in press].

2. Davey DA, MacGillivray I. The classification and definition of the hypertensive disorders of pregnancy. Am J Obstet Gynecol. 1988;158(4):892-8.

3. Stone P, Cook D, Hutton J, Purdie G, Murray H, Harcourt L. Measurements of blood pressure, oedema and proteinuria in a pregnant population of New Zealand. Aust N Z J Obstet Gynaecol. 1995;35(1):32-7.

4. Payne BA, Hutcheon JA, Ansermino JM, Hall DR, Bhutta ZA, Bhutta SZ, et al. A risk prediction model for the assessment and triage of women with hypertensive disorders of pregnancy in low-resourced settings: the miniPIERS (Pre-eclampsia Integrated Estimate of RiSk) multi-country prospective cohort study. PLoS Med. 2014;11(1):e1001589.

5. von Dadelszen P, Payne B, Li J, Ansermino JM, Broughton Pipkin F, Cote AM, et al. Prediction of adverse maternal outcomes in pre-eclampsia: development and validation of the fullPIERS model. Lancet. 2011;377(9761):219-27.

6. Magee LA, von Dadelszen P, Singer J, Lee T, Rey E, Ross S, et al. The CHIPS Randomized Controlled Trial (Control of Hypertension in Pregnancy Study): Is Severe Hypertension Just an Elevated Blood Pressure? Hypertension. 2016;68(5):1153-9.

7. Whelton PK, Carey RM, Aronow WS, Casey DE, Jr., Collins KJ, Dennison Himmelfarb C, et al. 2017 ACC/AHA/AAPA/ABC/ACPM/AGS/APhA/ASH/ASPC/NMA/PCNA Guideline for the Prevention, Detection, Evaluation, and Management of High Blood Pressure in Adults: A Report of the American College of Cardiology/American Heart Association Task Force on Clinical Practice Guidelines. Hypertension. 2018;71(6):e13-e115.

8. Reddy M, Rolnik DL, Harris K, Li W, Mol BW, Da Silva Costa F, et al. Challenging the definition of hypertension in pregnancy: a retrospective cohort study. Am J Obstet Gynecol. 2020;222(6):606 e1- e21.

9. Sutton EF, Hauspurg A, Caritis SN, Powers RW, Catov JM. Maternal Outcomes Associated With Lower Range Stage 1 Hypertension. Obstet Gynecol. 2018;132(4):843-9.

10. Hauspurg A, Sutton EF, Catov JM, Caritis SN. Aspirin effect on adverse pregnancy outcomes associated with stage 1 hypertension in a high-risk cohort. Hypertension. 2018;72(1):202-7.

**SCOPE office to complete**

**SCOPE RAF Number 4.38** **Approved**

**PI: L Magee PI email: laura.a.magee@kcl.ac.uk>**

| **Approval Process** | **Other** | **DATE** |
| --- | --- | --- |
| Received completed application |  | **3/11/2020** |
| Approved by Scientific Comm. | Yes, unconditional | **16/11/2020** |
| RAF resubmitted to Scientific Comm. for re assessment |  |  |
| Applicant notified of project final approval or decline |  |  |
| Project Agreement requested to be completed |  |  |
| Copy of Project Agreement to SCOPE office |  |  |
