# Supplementary material for: Diagnostic properties of differing BP thresholds for adverse pregnancy outcomes in standard-risk nulliparous women: A secondary analysis of SCOPE cohort data
Source: PLoS Med. 2025 Jan 22;22(1):e1004471. doi: 10.1371/journal.pmed.1004471 (PMC11798451; doi:10.1371/journal.pmed.1004471)
Supplement: S2 Table — (DOCX) [file pmed.1004471.s004.docx]

**S2 Table -** Adjusted RISK RATIOS for adverse pregnancy outcomes, according to the 2017 ACC-AHA BP categories (mmHg) based on BPs at 14-16 weeks*

|  | **Antenatal BP (mmHg)ł** | | | |
| --- | --- | --- | --- | --- |
|  | **Normal** | **‘Elevated BP’** | **‘Stage 1 HTN’** | **‘Stage 2 HTN’ (Non severe)** |
|  | **<120/<80** | **120-129/<80** | **130-139/80-89** | **140-159/90-109** |
|  | (N=4855) | (N=443) | (N=274) | (N=25) |
| **PPH >1L** | | | | |
| Event rate (n/N) | 188/4087 | 21/398 | 18/231 | 0/22 |
| aRR [95% CI] | 1.00 Ref | 0.97 (0.61, 1.52) | 1.27 (0.78, 2.06) | 0 (0,0) |
| **Preterm birth** | | | | |
| Event rate (n/N) | 281/4855 | 31/443 | 25/274 | 5/25 |
| aRR [95% CI] | 1.00 (Ref) | 1.14 (0.79, 1.63) | 1.48 (0.99, 2.22) | **3.19 (1.44, 7.03)** |
| **Birthweight <10^th^ centile** | | | | |
| Event rate (n/N) | 515/4855 | 55/443 | 48/274 | 6/25 |
| aRR [95% CI] | 1.00 (Ref) | 1.09 (0.83, 1.42) | 1.52 (1.15, 2.01) | **2.04 (1.01, 4.1)** |
| **Neonatal unit admission** | | | | |
| Event rate (n/N) | 534/4855 | 62/443 | 44/274 | 7/25 |
| aRR [95% CI] | 1.00 (Ref) | 1.13 (0.88, 1.45) | 1.32 (0.98, 1.77) | **2.15 (1.11, 4.15)** |
| **Preeclampsia** | | | | |
| Event rate (n/N) | 202/4850 | 45/443 | 25/274 | 6/25 |
| aRR [95% CI] | 1.00 (Ref) | **1.84 (1.33, 2.55)** | **1.66 (1.09, 2.55)** | **3.68 (1.75, 7.75)** |
| **Gestational hypertension or preeclampsia** | | | | |
| Event rate (n/N) | 202/4855 | 45/443 | 25/274 | 6/25 |
| aRR [95% CI] | 1.00 (Ref) | **1.91 (1.58, 2.31)** | **2.39 (1.95, 2.92)** | **2.9 (1.83, 4.61)** |

*aRR – adjusted risk ratio, BP – blood pressure, PPH – post partum haemorrhage, HTN – hypertension, CI – confidence interval, ACC (American College of Cardiology), AHA (American Heart Association)*

** As assessed at outpatient antenatal visits or medical assessment unit visits prior to the delivery admission. BP was categorized according to the 2017 ACC/AHA criteria as follows: ‘Normal’ (sBP <120mmHg and dBP <80mmHg), ‘Elevated BP’ (sBP 120-129mmHg but dBP <80mmHg), ‘Stage 1 hypertension’ (sBP 130-139mmHg and/or dBP 80-89mmHg), and ‘Stage 2 hypertension’ (sBP ≥140mmHg and/or dBP ≥90mmHg). RRs were adjusted for maternal age, body mass index at booking, ethnicity, smoking status, and alcohol use.*
