# Supplementary material for: Diagnostic properties of differing BP thresholds for adverse pregnancy outcomes in standard-risk nulliparous women: A secondary analysis of SCOPE cohort data
Source: PLoS Med. 2025 Jan 22;22(1):e1004471. doi: 10.1371/journal.pmed.1004471 (PMC11798451; doi:10.1371/journal.pmed.1004471)
Supplement: S3 Table — (DOCX) [file pmed.1004471.s005.docx]

**S3 Table:** Sensitivity, specificity, and likelihood ratios for SCOPE outcomes according to BP thresholds set by the 2017 ACC/AHA BP categories applied to BPs at 14-16 weeks gestation*

|  | **Antenatal BP (mmHg)*** | | | | |
| --- | --- | --- | --- | --- | --- |
|  | **Normal** | **‘Elevated BP’** | **‘Stage 1 HTN’** | **‘Stage 2 HTN’**  **(Non-severe)** | **‘Stage 2 HTN’**  **(Severe)** |
|  | **<120/<80** | **120-129/<80** | **130-139/80-89** | **140-159/90-109** | **≥160/ ≥110** |
|  | (N=4855) | (N=443) | (N=274) | (N=25) | (N=0) |
| **PPH >1L** | | | | | |
| Event rate (n/N) | *Ref* | 39/651 | 18/253 | 0/22 | - |
| Sensitivity [95% CI] | *Ref* | 0.17 (0.13, 0.23) | 0.08 (0.05, 1.12) | 0.00 (0.00, 0.02) | - |
| Specificity [95% CI] | *Ref* | 0.86 (0.85, 0.87) | 0.95 (0.94, 0.95) | 1.00 (0.99,1.00) | - |
| LR positive [95% CI]ł | *Ref* | 1.25 (0.93, 1.68) | 1.51 (0.95, 2.39) | 0 (0, 0) | - |
| LR negative [95% CI]ł | *Ref* | 0.96 (0.90, 1.02) | 0.97 (0.93, 1.01) | 1.00 (1.00, 1.01) | - |
| **Preterm birth** | | | | | |
| Event rate (n/N) | *Ref* | 61/742 | 30/299 | 5/25 | - |
| Sensitivity [95% CI] | *Ref* | 0.18 (0.14, 0.22) | 0.09 (0.06, 0.12) | 0.01 (0.00, 0.03) | - |
| Specificity [95% CI] | *Ref* | 0.87 (0.86, 0.88) | 0.95 (0.94, 0.95) | 1.00 (0.99,1.00) | - |
| LR positive [95% CI]ł | *Ref* | 1.38 (1.08, 1.75) | 1.71 (1.19, 2.46) | 3.84 (1.45, 10.17) | - |
| LR negative [95% CI]ł | *Ref* | 0.94 (0.90, 0.99) | 0.96 (0.93, 0.99) | 0.99 (0.98, 1.00) | - |
| **Birthweight <10^th^ centile** | | | | | |
| Event rate (n/N) | *Ref* | 109/742 | 54/299 | 6/25 | - |
| Sensitivity [95% CI] | *Ref* | 0.17 (0.15, 0.21) | 0.09 (0.07,0.11) | 0.01 (0.00, 0.02) | - |
| Specificity [95% CI] | *Ref* | 0.87 (0.86, 0.88) | 0.95 (0.94, 0.96) | 1.00 (0.99,1.00) | - |
| LR positive [95% CI]ł | *Ref* | 1.37 (1.14, 1.65) | 1.76 (1.32, 2.33) | 2.52 (1.01, 6.28) | - |
| LR negative [95% CI]ł | *Ref* | 0.95 (0.91, 0.98) | 0.96 (0.94, 0.99) | 0.99 (0.99,1.00) | - |
| **Neonatal unit admission** | | | | | |
| Event rate (n/N) | *Ref* | 113/742 | 51/299 | 7/25 | - |
| Sensitivity [95% CI] | *Ref* | 0.17 (0.15,0.21) | 0.08 (0.06,0.10) | 0.01 (0.00,0.02) | - |
| Specificity [95% CI] | *Ref* | 0.87 (0.86,0.88) | 0.95 (0.94,0.96) | 1.00 (0.99,1.00) | - |
| LR positive [95% CI]ł | *Ref* | 1.37 (1.14,1.65) | 1.57 (1.18,2.10) | 2.98 (1.25,7.10) | - |
| LR negative [95% CI]ł | *Ref* | 0.95 (0.91,0.98) | 0.97 (0.95,0.99) | 0.99 (0.98,1.00) | - |
| **Preeclampsia** |  |  |  |  |  |
| Event rate (n/N) | *Ref* | 76/742 | 31/299 | 6/25 | - |
| Sensitivity [95% CI] | *Ref* | 0.27 (0.22,0.33) | 0.11 (0.08, 0.15) | 0.02 (0.01, 0.05) | - |
| Specificity [95% CI] | *Ref* | 0.87 (0.87,0.88) | 0.95 (0.94, 0.96) | 1.00 (0.99, 1.00) | - |
| LR positive [95% CI]ł | *Ref* | 2.18 (1.78, 2.68) | 2.21 (1.56, 3.14) | **6.04 (2.43, 1.50)** | - |
| LR negative [95% CI]ł | *Ref* | 0.83 (0.77, 0.89) | 0.94 (0.90, 0.98) | 0.98 (0.96, 1.00) | - |
| **Gestational hypertension or preeclampsia** | | | | | |
| Event rate (n/N) | *Ref* | 224/742 | 107/299 | 12/25 | - |
| Sensitivity [95% CI] | *Ref* | 0.30 (0.27, 0.33) | 0.14 (0.12, 0.17) | 0.02 (0.01, 0.03) | - |
| Specificity [95% CI] | *Ref* | 0.89 (0.88, 0.90) | 0.96 (0.95, 0.97) | 1.00 (1.00, 1.00) | - |
| LR positive [95% CI]ł | *Ref* | 2.80 (2.44, 3.21) | 3.61 (2.89, 4.52) | **6.01 (2.75, 13.12)** | - |
| LR negative [95% CI]ł | *Ref* | 0.78 (0.75, 0.82) | 0.89 (0.87, 0.92) | 0.99 (0.98, 1.00) | - |
| *ACC (American College of Cardiology), AHA (American Heart Association), BP (blood pressure), CI (confidence interval), FP (false positive), HTN (hypertension), LR (likelihood ratio)*  ** As assessed at outpatient antenatal visits or medical assessment unit visits prior to the delivery admission. Diagnostic test properties were calculated for the lower limit of each BP category and above, vs. BP values below the category. BP was categorized according to the 2017 ACC/AHA criteria as follows: normal (sBP <120mmHg and dBP <80mmHg), elevated (sBP 120-129mmHg but dBP <80mmHg), Stage 1 hypertension (sBP 130-139mmHg and/or dBP 80-89mmHg), and Stage 2 hypertension (sBP ≥140mmHg and/or dBP ≥90mmHg).*  *† +LR was calculated as sensitivity/(1-specificity) and -LR as (1-sensitivity)/specificity.* | | | | | |
